# Neuroprotection by Nerve Growth Factor (NGF) involves modulation of neuronal autophagy

**DOI:** 10.1186/2193-1801-4-S1-P7

**Published:** 2015-06-12

**Authors:** Anna Maria Colangelo, Michele Papa, Miluscia Berbenni, Marco Pizzocri, Vita M Ippolito, Valentina Riganti, Lilia Alberghina

**Affiliations:** Lab. of Neuroscience “R. Levi-Montalcini”, Dept. of Biotechnology and Biosciences, University of Milano-Bicocca, Italy; Lab. of Morphology of Neuronal Network, Dept. of Public Medicine, Second University of Napoli, Italy

**Keywords:** NGF, Alzheimer Disease, autophagy

Nerve Growth Factor (NGF) plays a key role in development and function of specific neuronal populations of the CNS. Decreased NGF availability is also responsible for neuronal vulnerability in neurodegenerative disorders, such as Alzheimer’s disease (AD). We used PC12 cells and primary neurons to investigate the potential role of NGF in modulating neuronal autophagy, a cellular process whose deregulation has been linked to the loss of neuronal. The mammalian target of rapamycin has been recently proposed as a therapeutic target for AD due to the role of the autophagy pathway in improving cognitive function by reducing Aβ and Tau pathology. We here show that NGF treatment induces LC3 conversion (LC3-I to LC3-II), a marker of autophagy, through the activation of AMP-activated protein kinase (AMPK). We show that NGF increases the autophagic flux (the dynamic process of autophagosome synthesis, delivery to the lysosome and degradation) as indicated by western blot and fluorescence microscopy analyses in the presence of autophagy inhibitors. These data were confirmed by RT-PCR array analysis and RNA interference-mediated knockdown of autophagy-related genes: Atg9b, Atg12 and AMBRA1, a positive regulator of the BECLIN 1-dependent program of autophagy. In addition, flow cytometry analysis by DCF-DA showed that treatment with autophagy inhibitors determined a strong increase of reactive oxygen species (ROS) followed by decreased cell survival. These changes were fully reversed by NGF treatment, suggesting its potential role in clearing dysfunctional mitochondria. This hypothesis was further confirmed by fluorescence microscopy studies using LC3 and Cox-IV antibodies, showing co-localization of autophagosomes and mitochondria. Overall these data identify a novel aspect of the neuroprotective function of NGF in promoting the clearance of dysfunctional mitochondria (or mitophagy), thus further supporting its therapeutic potential in neurodegenerative pathologies.

